# Mason type III fractures of the radial head: ORIF, resection or prosthetic replacement?

**DOI:** 10.1007/s12306-022-00745-y

**Published:** 2022-04-16

**Authors:** D. Scoscina, G. Facco, P. Luciani, N. Setaro, L. Senesi, M. Martiniani, A. P. Gigante

**Affiliations:** 1 Department of Clinical and Molecular Sciences, Università Politecnica Delle Marche, Via Tronto 10/a, 60020 Torrette Di Ancona, Italy; 2 Department of Orthopaedic and Trauma Surgery, Ospedali Riuniti, Ancona, Italy; 3 Clinic of Adult and Paediatric Orthopaedics, Azienda Ospedaliero-Universitaria, Ospedali Riuniti Di Ancona, Ancona, Italy

**Keywords:** Mason type III fractures, Radial head arthroplasty, Radial head resection, Radial head ORIF, Radial head fractures complications

## Abstract

**Purpose:**

This study focused on a comparison of mid-term clinical, functional and radiographic outcomes of adults treated by open reduction and internal fixation (ORIF), radial head prosthesis (RHP) and resection (RHR).

**Methods:**

The retrospective evaluation concerned 47 surgically treated patients after a mean follow-up of 53 months. All patients were grouped according to the surgical procedure performed: 15 in the RHP group, 16 in the ORIF group and 16 in the RHR group. At the follow-up, outcome assessment was based on radiographs, range of motion (ROM) and functional rating scores.

**Results:**

Patients treated by RHR had significantly higher mean age and shorter operation time than other two groups. Compared to ROM, flexion, extension and pronation were significantly worse in patients treated by ORIF than those in the RHP group and the RHR group. Supination was significantly better in the RHP group. However, no statistical differences were observed in functional rating scores among the three groups. Regarding complications, instability was the only cause of revision surgery in the RHP group and the RHR group. On the other hand, the ORIF group revision rate was 50% and secondary displacement was the most frequent cause of failure.

**Conclusion:**

The ORIF group did not show good results with greater elbow stiffness and higher revision rate than the other two techniques. RHR may be suitable for elderly patients with lower functional demands as it reported good clinical results and reduced operation time.

## Introduction

Radial head fractures (RHFs) constitute a significant portion of elbow traumatic injuries in adults. They represent one-third of elbow fractures and the 4% of all fractures [1, 2]. RHFs equally affect males and females, although patient age and mechanism of trauma may vary [3, 4]: It usually involves a fall on an outstretched hand with the wrist extended and the pronated forearm.

Modified Mason classification is the most accepted for articular fractures of radial head [1, 5, 6]. Mason type I are minimally or non-displaced radial head fractures; type II are marginal sector fractures with displacement; type III are comminuted fractures involving the whole radial head, while type IV indicates RHFs associated with elbow dislocation.

While modified Mason type I and II are treated conservatively or by open reduction and internal fixation (ORIF) [7, 8], the optimum surgical solution for modified Mason type III and IV fractures is still debated in the literature [9, 10], especially due to residual instability from this type of injury [11].

It is now commonly agreed that two surgical procedures should be preferred especially in young patients: ORIF or radial head prosthesis (RHP). Some authors suggest that ORIF should be attempted when anatomic reduction, restoration of congruity and early motion can be achieved [12–14]. However, RHP has obtained a large consensus in managing comminuted fractures. In a recent systematic review, Heijink et al. [15] reported satisfactory mid-term functional results using RHP for unreconstructible RHFs. Although Antuña et al. [16] published good long-term outcomes of primary radial head resection (RHR), many works have described complications in the treatment of complex fracture [17]. Currently, RHR is reserved for low demanding patients, without soft tissue and bony injuries associated or after failed alternative management [18].

The aim of our retrospective study was to compare mid-term clinical, functional and radiographic outcomes of Mason type III RHFs in adults treated by ORIF, RHP or RHR.

## Materials and methods

Fifty-two patients with isolated closed comminuted RHF were surgically treated at Clinical Orthopedics, University Politecnica delle Marche (AN), between January 2014 and October 2019. The research was conducted by using an Institutional Review Board-approved trauma database. Patients provided informed consent for the inclusion in this retrospective study and publication of anonymized data. At a later stage, the patients’ medical records, surgical procedures and radiographic images were collected.

**Fig. 1:**
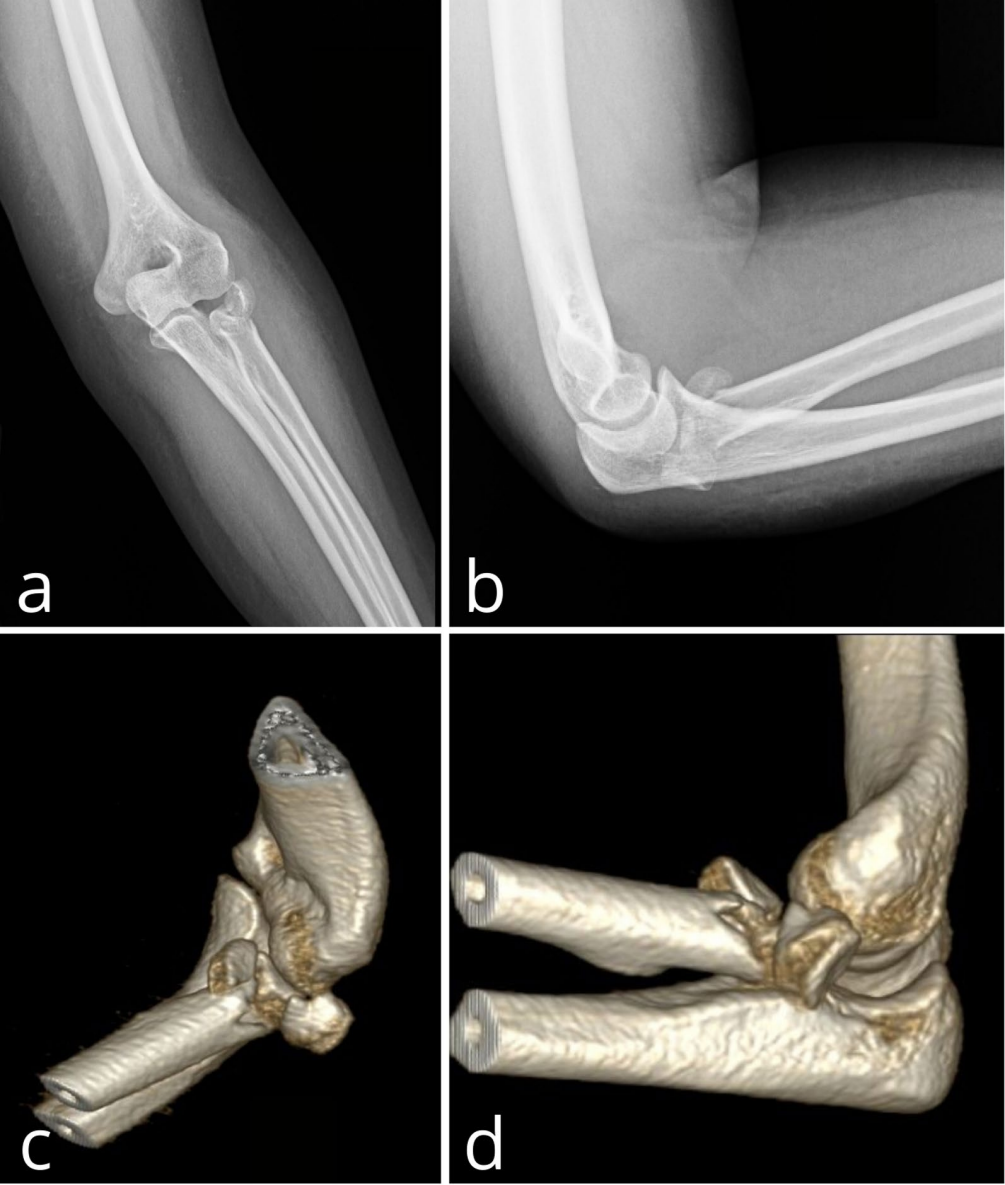
Anteroposterior (**a**) and lateral (**b**) X-ray and 3D CT (**c**, **d**) images of a patient with Mason type III radial head fracture

Inclusion criteria included: a diagnosis of Mason type III RHF based on the assessment of anteroposterior and lateral X-rays and computed tomography, (CT) scans including 3D reconstruction of the elbow joint (Fig. 1), skeletal maturity and a minimum 12-month follow-up. Exclusion criteria included: neurovascular injuries, patients with prior elbow fractures anamnesis and the ones treated after 10 days from their trauma, elbow dislocation and fracture(s) other than RHF. Patients with neurological diseases or systemic comorbidities that could compromise clinical results were also excluded.

Out of these 52 patients, 5 did not accept the clinical–radiological follow-up. In the end, our retrospective comparative study was based on 47 patients. All patients enrolled within our analysis were grouped according to the surgical procedure performed as follows: 15 in the RHP group, 16 in the ORIF group and 16 in the RHR group. The best treatment option depended on surgeon experience, degree of displacement, bone stock quality and patient’s functional requests. In our center, ORIF was preferred in young patients and resection in elderly patients. In minimally comminuted fractures with three or fewer articular fragments, ORIF was performed at first instance in young patients. In contrast, in fractures with four or more fragments with good bone stock, prosthetic replacement was the ideal treatment. If obtaining a stable synthesis with ORIF was impossible intraoperatively, the treatment was converted into a radial replacement in high functional request patients or fragments resection in low demanding patients.

### Surgical procedures

**Fig. 2:**
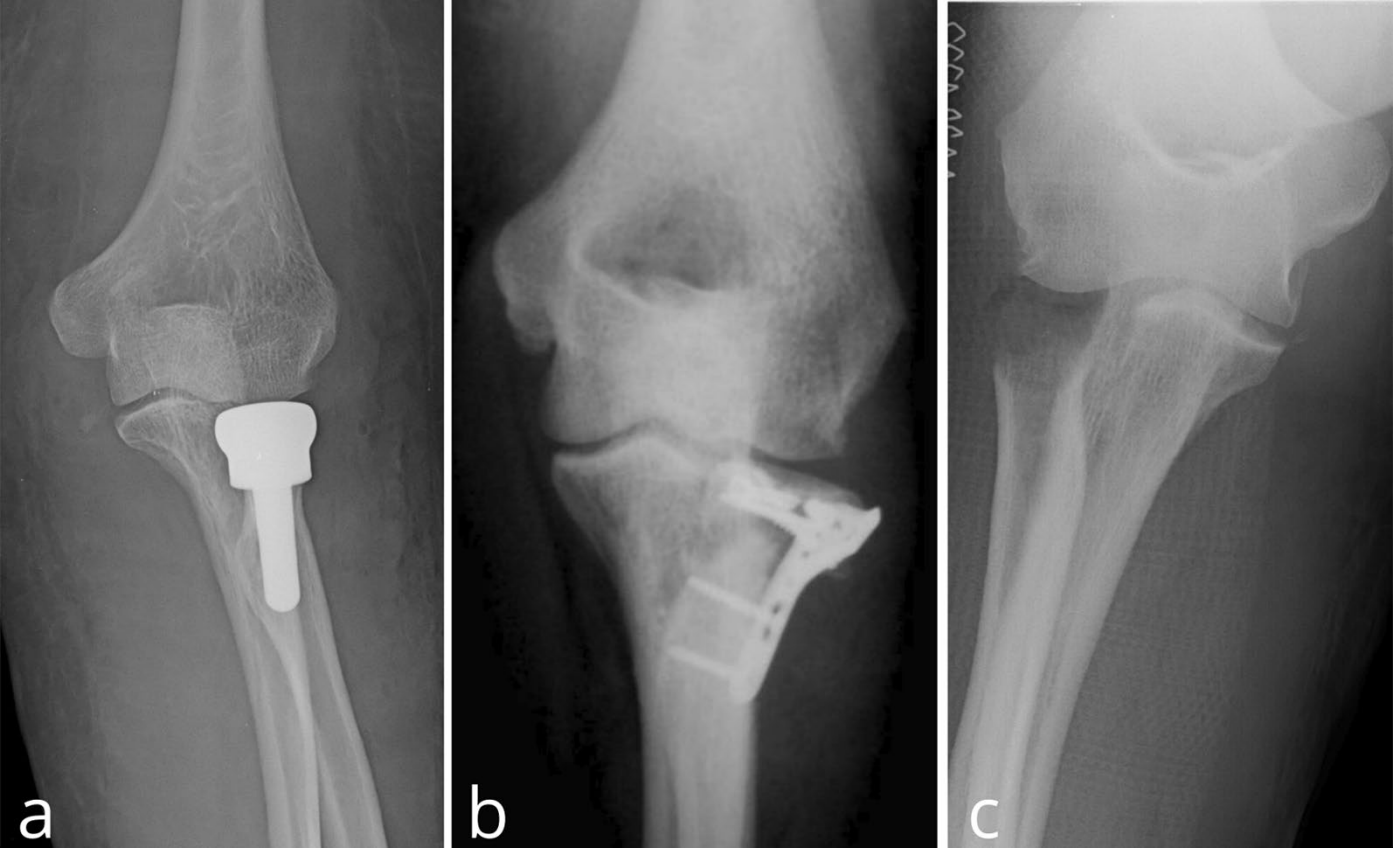
Postoperative anteroposterior X-ray of Mason type III radial head fracture treated with prosthetic replacement (**a**), open reduction and internal fixation (**b**) and resection (**c**)

A single expert upper-limb-specialized surgeon operated all patients. Prophylactic single-shot antibiotic was administered preoperatively. All patients were placed in the supine position with the forearm in abduction and pronated to protect the posterior interosseous nerve. Under brachial plexus block, a pneumatic tourniquet was applied. The surgical approaches were the same in the three groups. A Kocher’s posterolateral approach to the elbow joint was performed to dissect soft tissue into the intermuscular plane between the anconeus and the extensor carpi ulnaris. After transecting the annular ligament and the joint capsule along the previous split, the comminuted radial head was exposed. In the RHP group, all fragments were retrieved and assembled on table to select the type of radial head prosthesis, more closely restoring the native radial head size. Osteotomy of the radial neck and reaming of the proximal medullary canal of the radius were performed. After checking a good fit in the radial medullary canal and the capitulum humeri with trial prostheses and testing a full range of motion and the stability of the elbow joint, definitive stem and prosthetic head were inserted. In all cases, a modular unipolar cemented prosthesis (Zimmer Biomet) was implanted (Fig. 2a). In the ORIF group, the intraarticular hematoma was evacuated and the safe zone for proximal radioulnar articulation was identified. After the reduction in the fragments, a low-profile T-plates (Synthes) secured with appropriate screws were used for in situ fixation (Fig. 2b). In the RHR group, all fragments were removed, and the surface of bone stump was smoothed (Fig. 2c). Flexion–extension and rotational stability of the elbow joint was tested intraoperatively. Finally, capsule and annular ligament were repaired with nonabsorbable sutures.

Postoperatively, the patients were immobilized in a long-arm splint, with 90° of elbow flexion and neutral rotation. On postoperative day 2, patients of the RHP group and the RHR group initiated gradual active motion with the use of a hinged elbow brace, while in patients treated by ORIF an elbow plaster was applied for 1 week. Later, passive flexion–extension and prono-supination movement rehabilitation was allowed with the use of a hinged elbow brace. Active range of motion exercises began 4 weeks after surgery.

### Outcome evaluation

A demographic form reporting gender, date of trauma, age at surgery time, total months of follow-up and injured side was filled for each patient. Postoperative follow-up was conducted at 1, 3, 6 and 12 months, in addition to final visit. Physical examination included the measurement of active range of motion (AROM) as flexion, extension, pronation and supination with the use of a goniometer. Functional outcomes were assessed using the Broberg–Morrey elbow score [19] (0–100 points) and the Mayo Elbow Performance Score (MEPS) [20] (0–100 points). Subjective patient’s satisfaction was evaluated by using the QuickDASH [21]. Moreover, X-rays in standard anteroposterior and lateral views of the elbow joint were taken and analyzed. During the follow-up, clinical outcomes were evaluated by an expert orthopedic and radiographs were performed by two independent observers for the occurrence of any complications such as post-traumatic arthritis, osteolysis, loosening and implant dislocation, heterotopic ossification, avascular necrosis of the radial head, osseous nonunion and secondary displacement after internal fixation.

### Statistical analysis

The data were collected and organized using Excel (Microsoft, Redmond, WA, USA). Continuous variabilities were expressed as mean ± standard deviation (SD). Categorical variabilities were expressed in numbers and percentages. Differences between the three groups were compared by Mann–Whitney test and Fisher’s exact test when appropriate. Statistical analyses were performed using SPSS (version 21.0; IBM, Armonk, NY, USA). *p* values < 0.05 were assumed as statistically significant.

## Results

The patient demographics are reviewed in Table 1. The mean operation time in the prosthetic replacement and ORIF groups was significantly longer than in the resection group (*p* < 0.05). The mean follow-up periods were similar (*p* > 0.05), while the mean age of patients showed significant differences among the groups (*p* < 0.05). In fact, in patients treated with prosthesis the mean age was 53.9 ± 7.6, the ORIF group had a mean age of 41.7 ± 9.2, and the RHR group had a mean age of 64.5 ± 6.8.

**Table 1:** Patient demographic data

Group	Age at surgery (years)	% Female	Follow-up (months)	% Affecting dominant limb	Operation time (min)
RHP	53.9 ± 7.6	85	40.2 ± 8.9	70	70.5 ± 10.4
ORIF	41.7 ± 9.2	78	43.7 ± 11.1	73	75.3 ± 8.3
RHR	64.5 ± 6.8	83	45.2 ± 13.2	67	42.6 ± 5.2

RHP, radial head prosthesis; ORIF, open reduction and internal fixation; RHR, resection

When appropriate, data are reported as arithmetic means ± standard deviation

**Fig. 3:**
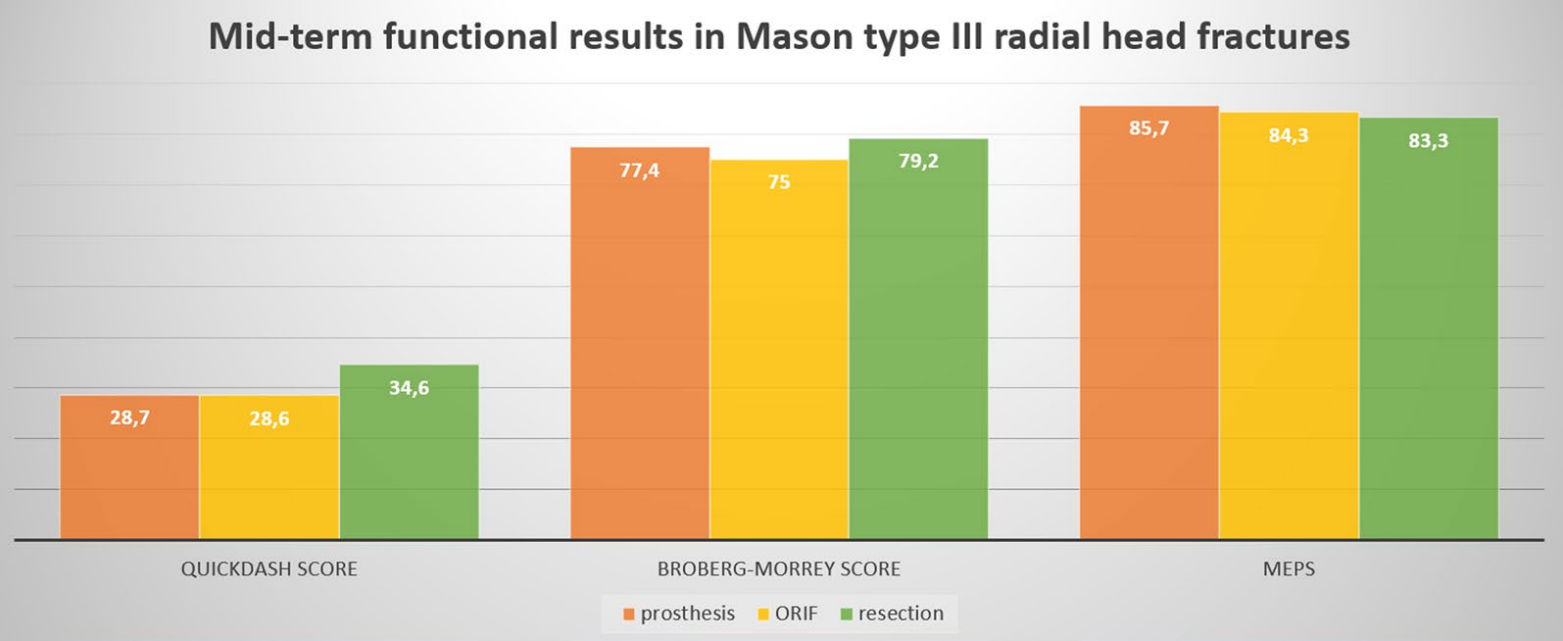
Mid-term functional results in Mason type III radial head fractures using QuickDASH, Broberg–Morrey and Mayo Elbow Performance Score (MEPS). Artwork created with Microsoft PowerPoint

Among elbow ROM measurements, active flexion, extension and pronation were significantly better in the RHP group and the RHR group than in the ORIF group, while in supination the prosthesis had better result than other surgical treatments. Regarding functional scores, no statistical differences were observed in QuickDASH score, Broberg–Morrey elbow score and MEPS between the three groups (Table 2, Fig. 3).

**Table 2:** Clinical results in three groups and *p* value in their comparisons

Variable	RHP group	ORIF group	RHR group	*p* value		
				RHP group vs ORIF group	RHP group vs RHR group	ORIF group vs RHR group
Active flexion	124.1 ± 16.4	110.6 ± 9.4	126.4 ± 11.4	**0.041**	0.97	**0.032**
Active extension	20.7 ± 11.1	30.1 ± 7.1	17.1 ± 9.1	**0.042**	0.53	**0.018**
Active pronation	68.1 ± 14.2	50.7 ± 18.3	68.7 ± 6.4	**0.032**	0.60	**0.042**
Active supination	77.3 ± 11.1	60.7 ± 14.3	62.1 ± 11.8	**0.028**	**0.031**	0.79
QuickDASH Score	28.7 ± 16.8	28.6 ± 13.5	34.6 ± 19.5	0.88	0.77	0.76
Broberg–Morrey Score	77.4 ± 15.3	75 ± 18.2	79.2 ± 21.3	0.65	0.33	0.57
MEPS	85.7 ± 17.5	84.3 ± 9.7	83.3 ± 18.3	0.40	0.90	0.83

RHP, radial head prosthesis; ORIF, open reduction and internal fixation; RHR, resection; DASH, disabilities of the arm, shoulder and hand; MEPS, Mayo Elbow Performance Score

When appropriate, data are reported as arithmetic means ± standard deviation. Bold value indicates statistical significance of *p* < .05

**Fig. 4:**
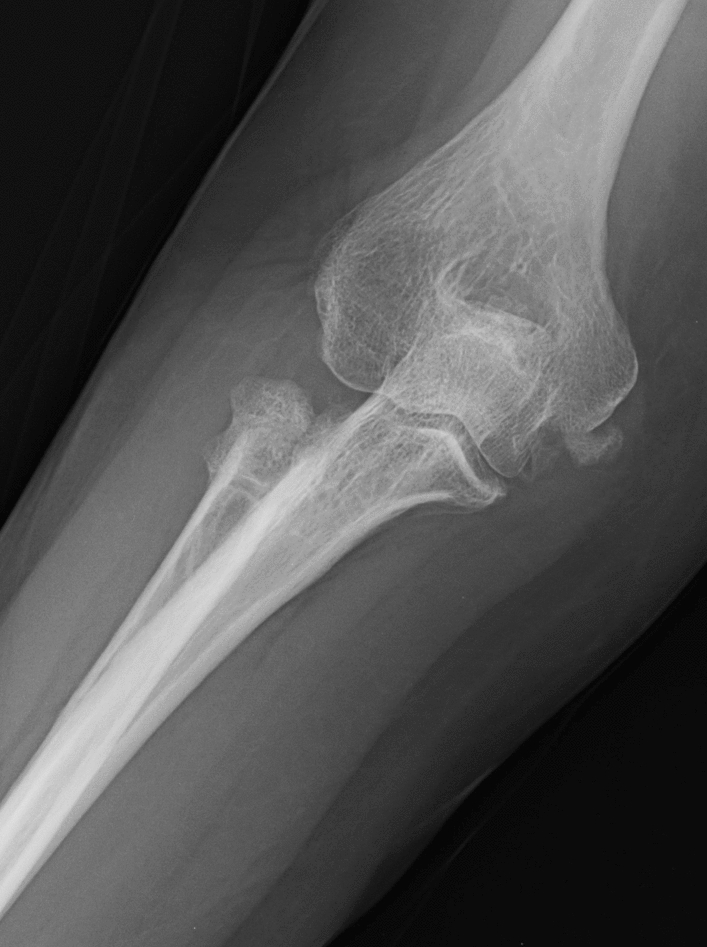
Six-month follow-up X-ray showing an ossification on the origin of the medial collateral ligament in the RHR group

With regards to complications (Fig. 4), in RHP, there were two cases of heterotopic periprosthetic ossification and one case of osteolysis, but additional surgery was not necessary. Two cases with instability of the prosthesis required implant revision. In the ORFI group, there were four cases of secondary displacement, two of nonunion and two of hardware breakage; in all these cases, a revision to RHP was performed. Finally, in the RHR group three cases with instability to stress in valgus and three cases of heterotopic ossification were observed. In two cases of instability, a revision to RHP was performed. There were no cases of postoperative infection, overstuffed and nerve palsy in the three groups (Table 3). The revision rate in the prosthetic replacement group and in the resection group was significantly lower than that in the ORIF group (*p* < 0.05).

**Table 3:** Complications and their treatments

Group	Complication	Number (%)	Treatment
RHP	Instability	2 (13.3%)	Replacing components
	Heterotopic ossification	2 (13.3%)	Observation
	Osteolysis	1 (6.6%)	Observation
ORIF	Secondary displacement	4 (25%)	RHP
	Nonunion	2 (12.5%)	RHP
	Hardware breakage	2 (12.5%)	RHP
	Heterotopic ossification	4 (25%)	Observation
RHR	Instability in valgus	3 (18.7%)	2 patient: RHP; 1 patient: observation
	Heterotopic ossification	3 (18.7%)	Observation

RHP, radial head prosthesis; ORIF, open reduction and internal fixation; RHR, resection

## Discussion

Radial head is a secondary valgus stabilizer of the joint, as demonstrated by Morrey et al. [22], as well as a contributor to posterolateral stability of the elbow. It is also involved in transferring 60% of axial load force through the elbow during flexion. Radial head stabilizing function becomes relevant in Mason type III RHFs associated with ligamentous injuries where instability is a complication such as in complex elbow dislocation, terrible triad injury and Essex–Lopresti fracture [23]. Operative treatment is recommended for Mason type III fractures, although the optimum surgical solution remains still object of debate [9]. The current study analyzed mid-term outcomes of 47 patients with isolated Mason type III RHFs treated with the following techniques: RHP, ORIF and RHR. In the literature, there are several studies that try to carry out which is the best technique, but none seems to be more efficient compared to the others.

Sun et al. [24] performed a meta-analysis comparing ORIF versus RHP treatment for Mason type III RHFs. The authors found higher satisfaction rate, better Broberg–Morrey elbow score and MEPS results, shorter operation time, lower incidence of bone nonunion or absorption and internal fixation failure in patient treated with RHP. In our study, the satisfaction rate and the operation time are similar between the two techniques although active flexion, extension, pronation and supination are better in the RHP group. Although patients of the ORIF group were about 10 years younger than ones of the RHP group, they developed greater stiffness.

Prior to the introduction of low-profile implants for ORIF and RHP, the best option of treatment in Mason type III RHFs was RHR [25]. Antuña et al. [16] published satisfactory long-term outcomes of RHR in RHFs occurred in 26 young patients. Their data did not show any postoperative complications. MEPS resulted 95 points and DASH 6 points.

Comparing RHR and RHP, Lópiz et al. [26] in their retrospective study concluded that RHR had better functional results and lower complications than RHP. In our study, we obtained better results with statistical significance only in supination movement in RHP (*p* = 0.031). Clinically, patients in both groups achieved satisfactory results at the functional scores, without statistically significant differences (*p* > 0.05), despite the younger age of the RHP group (mean age of 53.9 vs 64.5 years). Furthermore, the operation time of RHR was shorter than that of the other two groups. Conversely, comparing outcomes of ORIF and RHR, Ikeda et al. [27] recommended ORIF procedure because of stiffness and worst functional scores in the RHR group.

Comparing the three different treatments, Zwingmann et al. [28] analyzed 33 studies and outcomes in 302 patients. Unlike our study, they reported that ORIF obtained a success rate of 92% and has proved superior to other techniques although the results were not statistically significant (*p* = 0.266). Our study showed that at mid-term follow-up, RHP obtained better results in ROM evaluation, while ORIF showed the worst results between the three techniques. Despite this, also in our study the comparison of functional scores results did not show the superiority of a technique compared to the other (*p* > 0.05).

Heijink et al. [15] evidenced revision surgery in 8% of RHP. The main RHP complications were: osteolysis, subluxation, loosening, overstuffing, infection, stiffness, prosthetic stem fracture, lateral elbow pain, malposition and dissociation of the prosthesis. In the RHP group, we observed 33% of complications, but only the two cases of instability (13.3%) required revision surgery. Many works have described complications in the treatment of complex fractures with RHR, such as longitudinal instability with proximal migration of radius, elbow dislocation, increased valgus angle, humeroulnar osteoarthritis, lack of grip strength and ulnar neuropathy [17, 22]. Nevertheless, in our series only two patients (12.5%) treated with RHR required a revision to prosthesis for instability to stress in valgus. Also, ORIF may present complications such as nonunion and osteoarthritis [29]. In our ORIF series, many complications have occurred and the 50% of patients underwent a prosthetic revision. Secondary displacement was the main cause of revision as these are multifragmentary fractures very difficult to synthesize. Therefore, the present study reported a higher surgical revision rate for patients treated with ORIF compared to the ones treated with RHP and RHR.

The associated lesions (medial and lateral collateral ligaments, capsule, lateral and medial epicondylar muscles) are an important factor for treatment choice. It is necessary to consider primary and secondary elbow stabilizers and repair them in order to obtain good results and avoid instability [30].

The limitations of this study can be summarized as follows: (1) It was a retrospective and non-randomized study; (2) the small number of patients enrolled for each group; (3) only patients that underwent at the first surgery have been included; (4) the follow-up was not accepted by all patients and (5) was only mid-term. Further, randomized prospective studies with higher number of patients enrolled and longer follow-up, considering not only the first surgery but also hardware removal, when indicated, would be useful to define the *best technique* in Mason type III RHFs.

## Conclusion

In our experience, the prosthetic replacement of the radial head in isolated Mason type III RHFs has given better results on a clinical level, along with satisfaction among treated patients. Therefore, RHP may be preferable in young patients. Resection group has given good results too, with lower operation time than other techniques, whereas supination is reduced compared to RHP. For this reason, RHR is more suitable for older patients who do not have high functional demands. In ORIF group, outcomes are affected by the high comminution of the fractures and the relative risk of devascularization, leading to increased elbow stiffness and a high revision rate. Despite all, Broberg–Morrey elbow score, Mayo Elbow Performance Score and QuickDASH score gave suitable outcomes in all patients’ groups.

## Data Availability

Not applicable.
